# Awareness and Attitude of the General Public Toward HIV/AIDS in Coastal Karnataka

**DOI:** 10.4103/0970-0218.62580

**Published:** 2010-01

**Authors:** B Unnikrishnan, Prasanna P Mithra, Rekha T, Reshmi B

**Affiliations:** Department of Community Medicine, Kasturba Medical College, Mangalore, India; 1 Department of Health Information Management, Manipal College of Allied Health Sciences, Manipal, India

**Keywords:** AIDS, attitude, awareness, HIV, stigma

## Abstract

**Objective:**

To assess the awareness and attitude of the general public toward people living with HIV/AIDS (PLWHA) in Mangalore, a city in Coastal Karnataka.

**Design:**

Community-based cross-sectional study.

**Materials and Methods:**

The study population included 630 individuals aged 18 years and above. The information was collected using a semi structured pre-tested questionnaire. The questionnaire consisted of 24 questions regarding awareness of the modes of transmission of HIV/AIDS (nine questions) and questions to assess the attitude toward People Living With HIV/AIDS (PLWHA) (15 questions). Statistical package SPSS version 11.5 was used, Chi-square test was conducted and *P* < 0.05 was considered as statistically significant.

**Results:**

About one-third of the study population thought that one could get infected by merely touching an HIV positive individual. Approximately 45% stated that they would dismiss their maid on finding out her HIV positive status. About 54% were willing to undergo the HIV test. The respondents with less than secondary school education had a discriminatory attitude toward HIV positive people, with regard to them deserving to suffer, dismissing a HIV positive maid, hesitating to sit next to a HIV positive person in the bus, divorcing the infected spouse, and willingness to get tested for HIV, which was found to be statistically significant.

**Conclusion:**

Stigma among the general public was mostly due to fear of contracting the illness. Stigma does exist to significant degrees among the educated people, which was suggested by about 45% of the participants being willing to undergo the HIV test. There is a need for greater attempts toward making information regarding HIV/AIDS available to every individual of the society.

## Introduction

Acquired Immunodeficiency Syndrome (AIDS) is one of the most dreaded entities that modern medicine has ever had to tackle. Adult HIV prevalence in India is approximately 0.36%, which corresponds to an estimated 2 to 3.1 million people living with HIV in the country.(1)

Karnataka is one of the high HIV-prevalent states in India. According to the HIV Sentinel surveillance system data of 2007, HIV prevalence among pregnant women receiving antenatal care in Karnataka was 0.5% and those attending STD clinics was 8.4%.(2)

HIV-related stigma and discrimination remains an enormous barrier to effectively fighting the HIV and AIDS epidemic. Stigma is increased when the disease concerned is thought to be acquired entirely by the patient's fault, for example, immoral behavior.(3) Stigma and discrimination can result in People Living With HIV/AIDS (PLWHA) being shunned by family and the community, poor treatment in healthcare and educational settings, an erosion of rights, and psychological damage.

Stigmatization would make people hesitant to get the test done, therefore, more PLWHA are unaware that they are suffering from HIV/AIDS, and are thereby putting his/her sexual partners and/or needle sharers at risk of getting infected, due to lack of precautionary measures.(4)

There are several reasons for the stigma toward PLWHA among the general population, one of them could be inaccurate information about the transmission of HIV; creating irrational behavior and misperceptions of personal risks.(4)

Mangalore where the study was undertaken is the chief port city of Karnataka state, it is the administrative headquarter of the Dakshina Kannada District, has a population of 3.9 lakhs as per 2001 census, has an education rate of 83%, significantly higher than the national average of 59.5%. This area has also a favorable sex ratio of 1022.(5) According to the District Level Household and Facility Survey 3, school enrollment for both boys and girls in this district was 100%.(6)

Thus this study was carried out to assess the level of awareness among the general public about HIV/AIDS, along with their attitude toward PLWHA. Based on the findings, we needed to come up with suitable strategies to correct the misconceptions by Information, Education, and Communication (IEC) activities.

## Materials and Methods

A community-based, cross-sectional study was conducted in Mangalore among 630 participants over a period of three months (January to March 2007).

The calculated sample size for this study was 576; based on the anticipated proportion of awareness among the general population as 40%, with relative precision of 10%, and a Confidence Interval of 95%. After adding the 10% non-response error, the final sample size was 630.

The study area consisted of 60 wards with a total population of 0.398 million. People in the age group of 18 years and above were selected as the study population. Ten wards were selected out of the 60 wards by the simple random sampling method. The eligible populations of selected wards were listed, and a population proportionate to the sample size was calculated from each of the 10 selected wards. Selection of the households was done using convenience sampling. The information was collected using a semi-structured, pre-tested questionnaire. The questionnaire consisted of 24 questions with regard to the awareness of the modes of transmission of HIV/AIDS (nine questions) and questions to assess the attitude toward PLWHA (15 questions). During home visits the purpose and nature of the study was explained to the people and informed consent was obtained. On obtaining their consent, the investigator conducted a face-to-face interview to fill the questionnaire.

Data was analyzed using SPSS Version 11.5. Chi-square test was used to compare the awareness and attitude toward PLWHA across the educational category and p value <0.05 was considered statistically significant. The educational categories were defined as below secondary school (eighth standard) and secondary school and above.

## Results

The demographic profile of the study population is as shown in Table 1. Majority (62%) of the participants were aged between 18 and 29 years. Males (63%) outnumbered the females. Overall, 89.1% of the participants had an education of secondary school and above.

**Table 1:** Demographic profile of study population (n = 630)

Demographic variable	No.	Percentage
Age group (Years)		
<20	120	19.1
20–29	268	42.5
30–39	102	16.2
40–49	080	12.7
≥50	060	09.5
Gender		
Male	397	63.0
Female	233	37.0
Marital status		
Single	374	59.3
Married	252	40.0
Divorced	004	00.7
Occupation		
Government service	253	40.2
Business	096	15.3
Student	180	28.6
Housewife	037	05.8
Professional	021	03.3
Others (including unemployed)	043	06.8
Education		
Secondary or below	069	10.9
Higher secondary	107	17.0
Graduates	387	61.4
Postgraduates	067	10.7

The awareness of modes of transmission of HIV/AIDS is displayed in Table 2. More than 90% of the respondents appropriately reported that unsafe sex (98%), needle sharing (94%), and blood transfusion (97%) are possible ways of getting infected; 52% thought that HIV/AIDS does not spread via breast feeding. About one-third (34%) of the population were of the opinion that one can get infected by physical contact with a patient. It is reassuring to note that only a minority incorrectly stated that the disease spreads through mosquito bites (9 %), public toilets (7%), drinking from the glass an infected person has used (6%), and kissing on the cheeks (12%). On comparing the awareness of the respondents on the mode of spread of HIV and educational levels, more respondents with less than secondary education felt that HIV can spread by mosquito bite & kiss on the cheeks as compared to those with more than or equal to secondary level education and this difference was found to be statistically significant.

**Table 2:** Awareness of general public about mode of spread of HIV/AIDS (n = 630)

Mode of spread	<Secondary school No. (%)	≥Secondary school No. (%)	Total No. (%)	Chi square	*P* value
Unsafe sex	67 (97.1)	552 (98.4)	619 (98.2)	00.60	0.439
Mosquito bite	46 (66.7)	008 (01.4)	054 (08.6)	32.70	0.0001
Needle sharing	64 (92.8)	525 (93.6)	589 (93.5)	00.07	0.792
Public toilets	05 (07.2)	041 (07.3)	046 (07.3)	00.24	0.98
Blood transfusion	64 (92.8)	544(97.0)	608 (96.5)	03.24	0.072
Kiss on cheek	21(30.4)	057 (10.2)	078 (12.4)	23.28	0.0001
Breast feeding	10 (14.5)	295 (52.6)	305 (48.4)	35.70	0.0001
Sharing glass	07 (10.1)	032 (05.7)	039 (06.2)	02.09	0.149
Touching	28 (40.6)	185 (33.0)	213 (33.8)	01.59	0.208

Table 3 shows the attitude of the respondents toward PLWHA. Among the respondents, 81% were of the opinion that patients should not be isolated from society, 89% opined that infected children should attend regular schools, and 95% responded that they would not hesitate to sit next to a PLWHA in the bus. Sixty-one percent felt sympathetic toward PLWHA and 80% stated that they were willing to take an HIV/AIDS patient to the hospital from an accident site; 86% stated that they would not stop going to their usual grocery shop, if they found out that the owner was HIV positive. Only 12% stated that they would divorce their spouse if he/she turns out to be infected, 45% would dismiss their maid if she was HIV positive status, and 27% stated that they would be uneasy and apprehensive if their child's classmate had HIV/AIDS. Only about half the study population (54%) was willing to undergo the test for HIV/AIDS. The respondents with an educational level less than secondary school had a discriminatory attitude toward HIV positive people with regard to them deserving to suffer, dismissing a HIV-positive maid, hesitating to sit next to a HIV-positive person in the bus, divorcing the infected spouse, and willingness to get tested for HIV, which was found to be statistically significant.

**Table 3:** Attitude of the general public toward people with HIV/AIDS (n = 630)

Situation	< Secondary school No. (%)	≥Secondary school No. (%)	Total No. (%)	Chi square	*P* value
PLWHA are threat to society	10 (14.5)	056 (10.0)	066 (10.5)	01.33	0.248
Feel sympathetic to PLWHA	32 (46.4)	352 (62.7)	384 (61.0)	06.97	0.09
They deserve to suffer	09 (13.0)	140 (49.7)	149 (23.7)	04.83	0.03
Would you support your infected wards to get married	09 (13.0)	072 (12.8)	081 (12.9)	00.02	0.96
Would you stop shopping from a store if the owner is HIV positive	11 (15.9)	075 (13.4)	086 (13.7)	00.45	0.55
Would you feel uncomfortable if your child's classmate is HIV positive	21 (30.4)	150 (26.7)	171 (27.2)	00.43	0.51
Would you dismiss your HIV positive maid	46 (66.7)	238 (42.4)	284 (45.1)	14.59	0.0007
Would you hesitate to sit next to an HIV positive person	07 (10.1)	026 (33.3)	033 (05.2)	11.30	0.001
Would you help an accident victim if he is HIV positive	59 (85.5)	442 (78.8)	501 (79.5)	01.71	0.192
Would you divorce your infected spouse	14 (20.3)	063 (11.2)	077 (12.2)	04.70	0.03
Are you willing to get tested for HIV	26 (37.7)	314 (56.0)	340 (54.0)	08.27	0.04
Suppose you are HIV positive would you infect others	03 (04.3)	020 (03.6)	023 (03.7)	00.11	0.74
Should names of HIV patients made public	06 (08.7)	044 (07.8)	050 (07.9)	00.61	0.85
Should infected children be allowed in regular schools	60 (87.0)	498 (06.1)	558 (88.6)	00.20	0.65
Should HIV positive people be allowed to attend social functions	52 (75.4)	456 (81.3)	508 (80.6)	01.38	0.24

## Discussion

The awareness levels were satisfactory for all questions, with the right answer being given by a majority. However, only 48% knew breast-feeding to be a mode of transmission. Though 98% knew that HIV could spread through unsafe sex, and 96.5% knew that it could spread through blood, there was a small group (34%) who thought that HIV spreads by simply touching an infected individual. This proves that knowledge regarding how HIV/AIDS is NOT spread is less than the knowledge about how it is spread. Moreover, even when a person rightly knows that one will not get infected by a mere touch, the natural instinct of fear for one's safety still rules over the correct knowledge they have. Also, there was a disparity in the awareness regarding spread by mosquito bite, breastfeeding, and kiss on cheek as modes of transmission, between those who had only secondary school education or less and those who had studied further, which was found to be statistically significant.

It was also observed in this study that 11% of the participants considered PLWHA as a threat to society and about 61% felt sympathetic toward the infected. Among them, 54% were willing to undergo a test for HIV; when it was compared with the education levels it was found to be statistically significant. This could be attributed to the fear of being outcast from society on the occasion of being proved HIV positive. Almost half the population (45%) stated that they would dismiss their maid if she was found to be infected. This is difficult to judge as a stigma, because it could be due to fear arising out of concern for the safety of other members in the house. However, 86% of the respondents opined that they would continue to shop at a store if the owner was found to be HIV positive, the reason for the contradictory findings could be, that the risk depends on the kind of work the maid does in the household and the contact with the maid is on a regular basis and for a longer duration where as in case of the shop it would be on a once-in-a-while basis and there would not be intimate contact.

Almost 13% of the people opted to allow their son/daughter who was infected, to get married. This reflects that there is a need to increase the awareness regarding consequences of such practices and the moral duties one needs to pay attention to, in the event of your loved one becoming a victim of HIV/AIDS. The fact that more educated persons have a sympathetic attitude toward the HIV/AIDS patients is statistically significant. Also, a lesser percentage of those with higher education have stated that they would divorce their infected spouse. It is interesting to observe that although 89% have stated that infected children should be allowed in regular schools, 27% have stated they would be uncomfortable if such a child was in their own child's class. The possible explanation for this finding could be that even though they are sympathetic toward HIV infected children, when it comes to reality and to their own children they would not take the risk. However, a reasonable justification to such a response could be that the parent is concerned about the safety of his/her child, with regard to injuries or mishaps that can occur during school hours. This just proves the big difference that exists between wanting to reduce stigma and practicing a positive attitude to PLWHA in one's daily life.

One of the few similar studies done in the country is one from Andhra Pradesh (India), the findings being quite different.(7) They found that 51% of the general population wanted names of PLWHA to be made public, as compared to 8% in our study.

A study done among slum-dwellers in another metropolitan city of India (Chennai), showed that 67% males and 55% females were aware of the sexual mode of transmission, as compared to 98% in our study population.(8) In the same study, 45% males and 62% females thought AIDS could spread through mosquito bites, as compared to only 9% in our study.

Another study on awareness was done in China where they compared the responses from clients and from prostitutes. Interestingly, the prostitutes had a better knowledge regarding HIV transmission through needle sharing (77%) and from mother-to-child (75%) as compared to the 53% of clients, in both cases.(9) It is noteworthy that only 48% of our study population knew about HIV transmission through breast feeding, but 94% were aware of HIV transmission through needle sharing.

In another Indian study, 57% felt that people living with HIV/AIDS [PLWHA] should be isolated as compared to the 81% in our study who felt that PLWHA should NOT be stopped from attending public functions. Regarding the existence of a cure for AIDS, 14% of that study and 12% in our study thought that there is a cure for AIDS at present.(10)

Comparison with a similar study done in the US would be meaningful to understand cross-cultural differences in attitudes.(11) In that study, 40% responded that HIV transmission could occur through sharing a glass as compared to 6% from our study. A total of 19% responded that persons who acquired AIDS through sex or drug use have got what they deserved as compared to a greater number (24%) in our study.

Despite the moderately positive attitude of the general public toward PLWHA and satisfactory levels of awareness regarding the modes of transmission, they also exhibited certain misconceptions about the modes of transmission of HIV/AIDS. This being the result of a study on a literate population, we can visualize what the condition of the illiterate masses would be.

This study brings to light the need to plan and implement new strategies of educating the public about the modes of transmission of HIV. Special efforts should be made to make sure this information is available to the youth also, as they are exposed to drug abuse and unsafe sexual practices, due to peer pressure and an excessive adoption of western culture. This can be implemented by integrating relevant topics into the existing sex education syllabus, if any. If there is no sex education being given to adolescents of all socioeconomic backgrounds, this is a good incentive to start the same. They should be given access to a forum with trained persons, where they can discuss anything they need to clarify, especially those issues not discussed at home. If these trained persons are young adults rather than persons of their parents' age group, the two-way communication might become more effective and easier. Whether this is really true needs to be tested by using pilot studies. Also, parents need to be educated about the need to spend time with their children to impart good moral values and a non-secretive attitude to sex. Religious views regarding pre-marital and extra-marital sex can further help in teaching the need to avoid risky sexual behavior.

Regarding attitudes of the public toward PLWHA, interactive sessions and camps where HIV-infected persons share their experiences, could help give the public a better understanding of the lives of PLWHA.

Along with new action strategies, attention should also be given to better implementation of the existing programs to reduce stigma, increase awareness, and inculcate a more positive attitude toward PLWHA. These, along with the efforts of the healthcare professionals should provide an immense progress in the global fight against AIDS.
